# Predictors of COVID-19 vaccine uptake: an online three-wave survey study of US adults

**DOI:** 10.1186/s12879-024-09148-9

**Published:** 2024-03-12

**Authors:** Alistair Thorpe, Angela Fagerlin, Frank A. Drews, Holly Shoemaker, Federica S. Brecha, Laura D. Scherer

**Affiliations:** 1 Department of Population Health Sciences, Spencer Fox Eccles School of Medicine at University of Utah, Salt Lake City, UT USA; 2 Department of Applied Health Research, University College London, London, UK; 3 Salt Lake City VA Informatics Decision- Enhancement and Analytic Sciences (IDEAS) Center for Innovation, Salt Lake City, UT USA; 4 University of Utah College of Social and Behavioral Science, Salt Lake City, UT USA; 5 Department of Pediatrics, Columbia University, New York, NY USA; 6 Division of Cardiology, University of Colorado, School of Medicine, Aurora, CO USA; 7 Denver VA Center of Innovation, Denver, CO USA

**Keywords:** COVID-19, COVID-19 vaccines, Mass vaccination, Vaccine hesitancy, Vaccination coverage, Public health

## Abstract

**Background:**

To effectively promote vaccine uptake, it is important to understand which people are most and least inclined to be vaccinated and why. In this study, we examined predictors of COVID-19 vaccine uptake and reasons for non-vaccination.

**Methods:**

We conducted an online English-language survey study in December-2020, January-2021, and March-2021. A total of 930 US respondents completed all surveys. Multiple logistic regression models were run to test whether the early vaccine eligibility, demographic factors, and psychological factors predict getting at least one dose of a COVID-19 vaccination in January-2021 and in March-2021.

**Results:**

The proportion of respondents who received ≥ 1-dose of a COVID-19 vaccine increased from 18% (January) to 67% (March). Older age predicted vaccine uptake in January (OR = 2.02[95%CI = 1.14–3.78], *p* < .001) and March (10.92[6.76–18.05], *p* < .001). In January, additional predictors were higher numeracy (1.48[1.20–1.86], *p* < .001), COVID-19 risk perceptions (1.35[1.03–1.78], *p* = .029), and believing it is important adults get the COVID-19 vaccine (1.66[1.05–2.66], *p* = .033). In March, additional predictors of uptake were believing it is important adults get the COVID-19 vaccine (1.63[1.15–2.34], *p* = .006), prior COVID-19 vaccine intentions (1.37[1.10–1.72], *p* = .006), and belief in science (0.84[0.72–0.99], *p* = .041). Concerns about side effects and the development process were the most common reasons for non-vaccination. Unvaccinated respondents with no interest in getting a COVID-19 vaccine were younger (0.27[0.09–0.77], *p* = .016), held negative views about COVID-19 vaccines for adults (0.15[0.08–0.26], *p* < .001), had lower trust in healthcare (0.59[0.36–0.95], *p* = .032), and preferred to watch and wait in clinically ambiguous medical situations (0.66[0.48–0.89], *p* = .007).

**Conclusions:**

Evidence that attitudes and intentions towards COVID-19 vaccines were important predictors of uptake provides validation for studies using these measures and reinforces the need to develop strategies for addressing safety and development concerns which remain at the forefront of vaccine hesitancy.

**Supplementary Information:**

The online version contains supplementary material available at 10.1186/s12879-024-09148-9.

## Background

COVID-19 continues to pose a significant threat to public health. Widespread uptake of the multiple vaccines authorized by the U.S. Food and Drug Administration for use against COVID-19 represents the safest and most effective strategy for limiting the impact of the disease [1]. However, with the Centers for Disease Control and Prevention (CDC) COVID Data Tracker reporting that ~20% of adults in the United States have not received a primary dose of a COVID-19 [2], public hesitancy and refusal to get vaccinated remains a major challenge to realizing the full preventative health benefits of the authorized COVID-19 vaccines.

In order to effectively promote vaccine uptake, it is important to first understand which people are most and least inclined to be vaccinated and why. Over the course of the pandemic, research identifying important demographic (e.g., age, race, ethnicity, and education) [3, 4] and psychological factors (e.g., COVID-19 risk perceptions [3–6], belief in conspiracy theories [7], political affiliation [3, 5, 6, 8], exposure to misinformation [9], and trust in scientists [5, 10], and the government [10, 11]) associated with public attitudes and intentions towards COVID-19 vaccines has accumulated at a rapid rate. This research has been of great value to policy makers and health communicators aiming to develop strategies and interventions to address concerns about COVID-19 vaccines and promote vaccine uptake.

However, although attitudes and intentions towards vaccination are often useful predictors of actual vaccine uptake [12, 13] this relationship does not always hold true [14–18]. For example, it is well documented that many people who intend to receive an influenza vaccine ultimately do not go on to receive one [14, 16]. While evidence regarding the predictors of attitudes and intentions towards COVID-19 vaccination and characteristics associated with uptake has accumulated at a dramatic rate during the pandemic, there are fewer studies that have sought to predict actual uptake of COVID-19 vaccination. Among studies that have examined factors associated with COVID-19 vaccine uptake, most have tended to consider the influence of demographic factors such as age, gender, and socioeconomic status [19–21]. Only a minority have also considered potential psychological influences, with those that do focussing on one or two specific factors such as vaccine attitudes [22, 23], mistrust [24], and risk perceptions [23, 25]. As a result, there is a lack of data on the extent to which demographic and psychological factors, when considered together, predict actual uptake of COVID-19 vaccination. Furthermore, many cross-sectional studies of COVID-19 vaccine uptake may not capture changes in public behaviour across evolving periods of the pandemic.

Thus, data is therefore needed to identify attitudinal and sociodemographic factors that predict future vaccine uptake over time. The aim of the present study is to identify factors that predicted uptake of COVID-19 vaccination when vaccines first became available in January and March, 2021. In addition, we report the reasons given for not getting vaccinated by those who had not and did not intend to do so, following the rollout of the COVID-19 vaccines in the US in December, 2020.

We hypothesized that older age, living in a state with a greater proportion of people vaccinated, Veteran status, having a greater number of pre-existing health conditions, higher health literacy, higher numeracy,^1^ and being non-Hispanic White, would be associated with having received at least one dose of a COVID-19 vaccine in both January and March, 2021. Based on existing research on psychological factors associated with COVID-19 vaccine attitudes and intentions, we also expected that greater worry about COVID-19, greater COVID-19 risk perceptions, greater confidence in vaccines, greater intentions to get a COVID-19 vaccine, greater trust in health care, greater belief in science, less belief in conspiracies, more liberal political views, and medical maximizing would be associated with COVID-19 vaccine uptake.

## Methods

### Study design and population

Respondents were recruited and compensated by Qualtrics Online Panels for three nonprobability internet surveys as part of a longitudinal study conducted in December 2–27, 2020 (*n*_Veteran_=1060; *n*_nonVeteran_=1025), January 21-February 6, 2021 (*n*_Veteran_=746; *n*_nonVeteran_=511), and March 8–23, 2021 (*n*_Veteran_=688; *n*_nonVeteran_=387) [26]. Our sample size was determined a-priori to achieve a sample of 1,000 Veteran and 1,000 non-Veteran respondents for the first survey and to account for a 20% attrition rate for the 2nd and 3rd surveys, but did not include any formal power analysis. In past studies we have conducted on pandemic communication, a sample size of 1000 was more than sufficient to find clinically meaningful significant effects [27–30]. To meet the study inclusion criteria potential respondents were required to be 18 years or older, US residents, and have access to the internet. We implemented several strategies to ensure that the survey could not be completed by “bots” or cheaters including Google’s invisible reCAPTCHA, security scan monitor, preventing multiple submissions, and blocking search engines access (for further details see: https://www.qualtrics.com/support/survey-platform/survey-module/survey-checker/fraud-detection/).

Surveys were presented in English and administered online. This study was deemed exempt by the University of Utah and the Salt Lake City VA IRBs and follows the reporting guidelines of the American Association for Public Opinion Research (AAPOR). Invited participants first read a consent cover letter which stated that consent was indicated by completing the questionnaires.

### Procedure and measures

Over a four-month period (December, 2020 to March, 2021), respondents completed a three-wave survey study with the first survey (Wave 1) sent to respondents in December, 2020, the second (Wave 2) in January, 2021, and the third (Wave 3) in March, 2021. All three surveys are available on the project repository (https://osf.io/63gte/) and consisted of questions about respondents’ current behaviors, well-being, healthcare experiences, and attitudes regarding the COVID-19 pandemic [26]. Both the January and March surveys also contained short message-based experiments regarding the COVID-19 vaccines published elsewhere [31, 32]. All three surveys were developed by the study team, which includes psychologists and health services researchers with extensive experience with online survey methodology. We used validated measures and measures from prior studies where possible and only adapted or created new measures when absolutely necessary. Descriptions of all measures included in the analyses are available in the online supplementary materials.

*Primary outcome measure.* Self-reported vaccination status was measured using a single question with three options (0 = No; 1 = Yes, 1 dose; 2 = Yes, 2 doses) in January and March, 2021. As responses 1 and 2 indicated receiving at least one dose of a COVID-19 vaccine, they were considered vaccinated for analyses (0 = Not vaccinated; 1 = Vaccinated).

*Early vaccine eligibility*. Respondents’ age and the total number of pre-existing conditions [33] were included based on recommendation by the Centers for Disease Control and Prevention (CDC) for these populations to be offered vaccines first [34]. As the speed of vaccine distribution within each state may affect vaccine availability for those eligible we also included the proportion of each state that had received at least one dose of a COVID-19 vaccine (retrieved from publicly available data: https://www.kff.org/coronavirus-covid-19/issue-brief/state-covid-19-data-and-policy-actions/). Veteran status (0 = non-Veteran; 1 = Veteran) was also included given the involvement of the U.S. Department of Veterans Affairs in the distribution of COVID-19 vaccines following their authorization [35]. 

*Demographic factors*. We included respondents’ health literacy [36], numeracy [37, 38] and Race/Ethnicity (dummy coded as 0 = any other race/ethnicity; 1 = non-Hispanic White).

*Psychological factors*. We included respondents’ worries and risk perceptions about COVID-19, the Emory Vaccine Confidence Index [39], perceived importance of influenza and COVID-19 vaccines, COVID-19 vaccine intentions, trust in healthcare [40], (lack of) belief in science [41], belief in conspiracy theories [42], political views, and the single-item maximizer-minimizer elicitation question (the MM1; which measures preference for either waiting or taking action in medical situations where it is unclear whether action is needed) [43]. 

### Statistical analysis

All the analyses were conducted in R Studio Version 1.4.1106. We used the “psych” package to run bivariate correlations between our predictor variables and vaccine uptake. Using the “stats” package, we ran a multiple logistic regression model to test whether the early vaccine eligibility and demographic factors predict getting at least one dose of a COVID-19 vaccination in January 2021. Using a hierarchical approach, we then included the psychological factors to the original model. We then repeated this analytical approach with receiving at least one dose of a COVID-19 vaccine in March, 2021 as the dependent variable. Reported estimates are without adjustment for non-response bias with comparisons between respondents who completed all three surveys with those who completed only one or two surveys reported elsewhere [44]. No imputation methods were used to account for missingness as the overall rate of missingness across study variables was low (< 0.2%). Respondents who reported having received at least one dose of a COVID-19 vaccination in January 2021 were not included in the March 2021 analyses.

## Results

### Sociodemographic information

A total of 930 respondents completed all three surveys and were included in the analyses. Information on the 1,155 respondents who did not complete all three surveys is available on the project repository (https://osf.io/63gte/) (26, 44). The completion rate was 44% overall, 55% for Veterans, and 33% for non-Veterans. Respondents in our sample were generally older (median age ranged between 55 and 74 years old), male (735, 79%), non-Hispanic White (720, 77%), US Veterans (584, 63%), and with a median household income between $50,000-$99,999. Over half of respondents (440, 64%) reported having a pre-existing condition that made them more vulnerable to COVID-19; 186 respondents (27%) indicated that they did not have such a pre-existing condition and 67 respondents (10%) were not sure. In January, 165 respondents (18%) reported having received a COVID-19 vaccine; 160 (97%) of those were first doses and only 5 (3%) had received both doses. The number of respondents reported having been vaccinated increased to 620 (67%) in March with 206 (33%) first doses and 414 (67%) both doses. Full demographics are shown in Table 1.

**Table 1:** Respondent demographics overall and according to Veteran status

			Overall *(n = 930)*	Veteran *(n = 584)*	Non-Veteran *(n = 346)*
Age in yrs – no. (%)					
	18 to 34		37 (4)	0 (0)	37 (11)
	35 to 54		87 (9)	16 (3)	71 (21)
	55 to 74		591 (64)	390 (67)	201 (58)
	75 or older		213 (23)	176 (30)	37 (10)
	Did not respond		2 (< 1)	2 (< 1)	0 (0)
Gender – n (%)					
	Female		193 (21)	38 (7)	155 (45)
	Male		735 (79)	545 (93)	190 (55)
	Non-binary/Third gender or Transgender man/Transman		2 (< 1)	1 (< 1)	1 (< 1)
Race/Ethnicity – no. (%)					
	Non-Hispanic White		720 (77)	447 (77)	273 (79)
	Non-Hispanic Black		64 (7)	44 (8)	20 (6)
	Hispanic		92 (10)	61 (10)	31 (9)
	Asian/Asian American		26 (3)	9 (2)	17 (5)
	American Indian/Alaskan Native		4 (< 1)	4 (1)	0 (0)
	Native Hawaiian/Other Pacific Islander		2 (< 1)	2 (< 1)	0 (0)
	Another race		14 (2)	11 (2)	3 (1)
	Multiracial		8 (1)	6 (1)	2 (1)
Income – no. (%)					
	$0 - $49k		206 (22)	117 (20)	89 (26)
	$50K to $99K		362 (39)	232 (40)	130 (38)
	$100K or more		325 (35)	216 (37)	109 (32)
	Prefer not to say		37 (4)	19 (3)	18 (5)
Residence – no. (%)					
	Rural		151 (16)	96 (16)	55 (16)
	Small city (*< 100,000*)		159 (17)	101 (17)	58 (17)
	Suburban, near a large city		457 (49)	277 (47)	180 (52)
	Mid-sized city (*100,000–1 million)*		90 (10)	60 (10)	30 (9)
	large city (*> 1 million)*		70 (8)	47 (8)	23 (7)
	Other		3 (< 1)	3 (1)	0 (0)
Vaccination status in January 2021 – no. (%)					
	None		765 (82)	463 (79)	302 (87)
	One dose		160 (17)	118 (20)	42 (12)
	Two doses		5 (1)	3 (1)	2 (1)
Vaccination status in March 2021 – no. (%)					
	None (March)		310 (33)	146 (25)	164 (47)
	One dose (March)		206 (22)	128 (22)	78 (23)
	Two doses (March)		414 (45)	310 (53)	104 (30)

### Reasons for not getting a COVID-19 vaccine

In the March, 2021 survey, a total of 310 respondents (33% of the total sample) had not received a vaccine. Almost a quarter of those respondents (69, 22%) reported that they did not want to get one. Among the 69 unvaccinated respondents who did not want to receive a COVID-19 vaccine, concerns about possible side effects and the vaccine development process were the most frequently endorsed reason for not getting vaccinated (Table 2). Other reasons for not getting vaccinated included not believing COVID-19 poses a serious threat, personal beliefs (e.g., religious and philosophical) that conflicted with getting vaccinated, and distrust of the institutions involved with promoting vaccines (e.g., pharmaceutical companies and the government). A few respondents cited doubts about the efficacy of the vaccines and a very small proportion reported access issues (e.g., not having enough time or vaccines being unavailable) as reasons for not getting vaccinated.

**Table 2:** Reasons for not getting a COVID-19 vaccine among unvaccinated respondents who were not interested in getting a COVID-19 vaccine

		December 2020 (N = 69)	January 2021 (N = 69)	March 2021 (N = 69)
Safety – no. (%)				
	Concerns about side effects	24 (35)	14 (20)	20 (29)
	Concerns about vaccine development process	5 (7)	16 (23)	12 (17)
	Worried about getting COVID-19 from the vaccines	3 (4)	2 (3)	2 (3)
	I don’t like needles	4 (6)	1 (1)	2 (3)
Efficacy – no. (%)				
	Doubt vaccine efficacy	4 (6)	7 (10)	4 (6)
Low COVID-19 threat – no. (%)				
	I won’t get COVID-19 even if I don’t get the vaccine	4 (6)	5 (7)	5 (7)
	COVID-19 is not as serious as some people say	5 (7)	3 (4)	6 (9)
	I do not think I’ll get very sick if I get COVID-19	2 (3)	3 (4)	1 (1)
Personal beliefs – no. (%)				
	Against religious/philosophical beliefs	6 (9)	3 (4)	4 (6)
	Distrust of big Pharma/government	5 (7)	5 (7)	5 (7)
Other reasons – no. (%)				
	Already had COVID-19	1 (1)	3 (4)	2 (3)
	No specific reason/multiple reasons/Unsure	-	-	3 (4)
	Did not respond	5 (7)	5 (7)	-
	I plan to get the vaccine	-	1 (1)	1 (1)
	Access/Availability/Cost	-	1 (1)	-
	Medical reasons (e.g., allergies)	1 (1)	-	2 (3)

### Logistic regressions

In the regression models which only included the early vaccine eligibility and demographic factors, we found that older age (OR *=* 2.54[95%CI = 1.47–4.65], *p* = .001), the proportion of the state vaccinated (OR = 1.09[1.00–1.19], *p = *.041), increased number of pre-existing conditions (OR = 1.17[1.03–1.33], *p* = .034), and higher numeracy (OR = 1.59[1.30–1.97], *p* < .001) predicted vaccine uptake in January. Older age (OR = 9.10[6.01–14.03], *p* < .001), increased number of pre-existing conditions (OR = 1.19[1.04–1.35], *p* = .011), and higher numeracy (OR = 1.18[1.01–1.37], *p* = .035) were significant predictors of later vaccine uptake (in March).

**Fig. 1:**
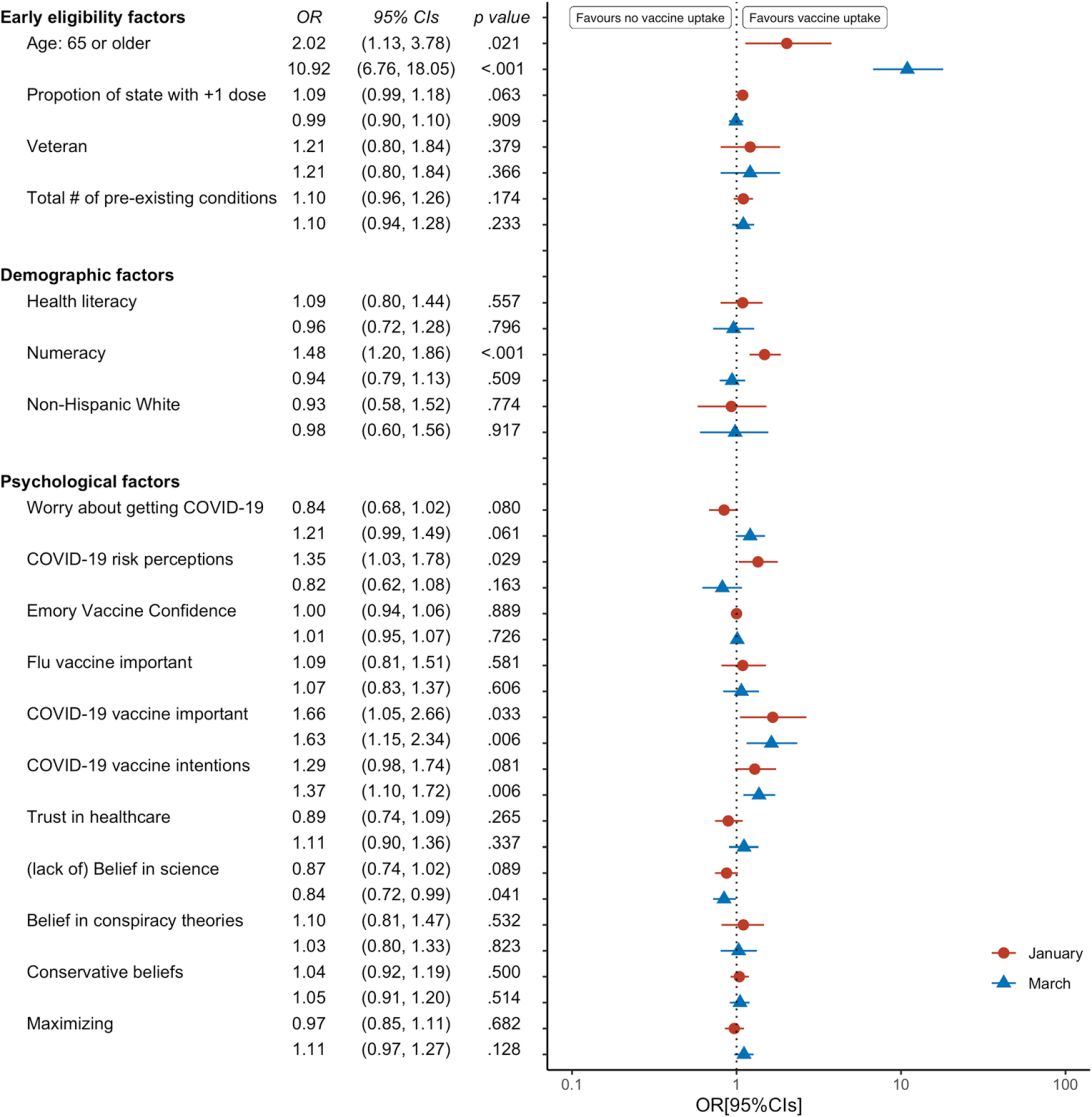
Odds ratios with 95% confidence intervals for predictors of respondents’ vaccination status in both January and March 2021. Reference categories were 64 or younger (for Age), non-Veteran (for Veteran), and any other Race/Ethnicity (for Non-Hispanic White)

After including the psychological variables, older age remained a predictor of vaccine uptake in both January and March (Fig. 1). Higher numeracy, higher COVID-19 risk perceptions, and believing that it is important for all adults to get the COVID-19 vaccine were also predictors of vaccine uptake in January. In March, believing that it is important for all adults to get the COVID-19 vaccine, prior intentions to get a COVID-19 vaccine, and general belief in science predicted vaccine uptake alongside older age.

**Fig. 2:**
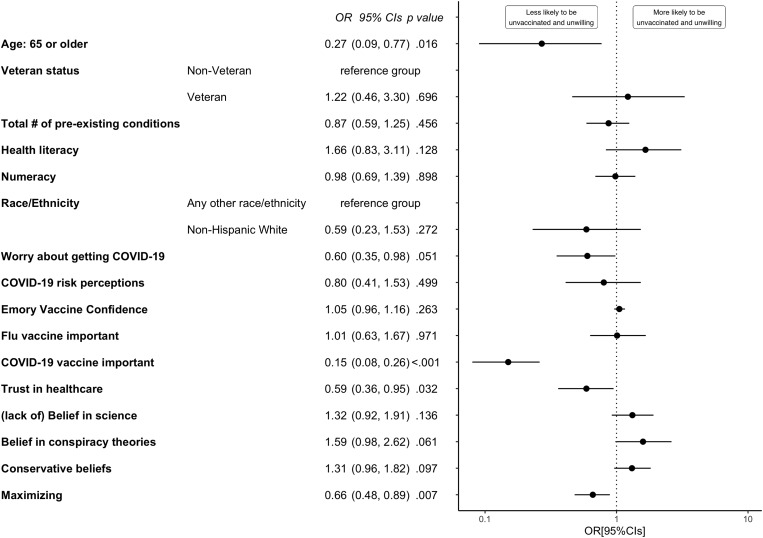
Odds ratios with 95% confidence intervals for predictors of being unvaccinated and not wanting a COVID-19 vaccine by March 2021 (*N* = 925, R^2^ Tjur = 0.63)

In a further exploratory analyses, we found that younger age (OR = 0.27[0.09–0.77], *p* = .016), believing it is not important for all adults to get a COVID-19 vaccine (OR = 0.15[0.08–0.26], *p* < .001), low trust in healthcare (OR = 0.59[0.36–0.95], *p* = .032), and preferring to watch and wait in medical situations where it is not clear whether or not medical action is necessary (OR = 0.66[0.48–0.89], *p* = .007), were significant predictors of being unvaccinated and not wanting to receive a COVID-19 vaccine by March 2021 (Fig. 2).

## Discussion

The aim of the present study was to identify key predictors of, and objections to, COVID-19 vaccine uptake using a nonprobability online longitudinal survey of US Veterans and non-Veterans between December 2020 and March 2021. Building on previous work, we considered a range of demographic and psychological factors that may be associated with COVID-19 vaccine uptake. Findings from our sample, revealed that older age, higher numeracy, higher COVID-19 risk perceptions, and positive attitudes towards COVID-19 vaccines were important predictors of early vaccine uptake (by January 2021). As the rollout progressed, the influence of numeracy and risk perceptions remitted and we found that only older age, positive attitudes towards COVID-19 vaccines, and intentions to receive a COVID-19 vaccine were significant predictors of later vaccine uptake (by March 2021).

Consistent with prior research [20, 21], older age was the strongest predictor of vaccine uptake for both timepoints, which reflects its emphasis as key criterion for early vaccine eligibility by the CDC [34]. In contrast, the proportion of other people within the state who had been vaccinated, Veteran status, and the total number of pre-existing conditions were not associated with COVID-19 vaccine uptake at either time points. The combination of numeracy, risk perceptions, and attitudes towards COVID-19 vaccines as predictors of early vaccine uptake supports prior research demonstrating that assessment of the risks and benefits offered by vaccination as well as the threat of the disease that the vaccine protects against have a substantial influence on whether or not someone is likely to get vaccinated [45, 46]. While clear communication about the risks and benefits associated with the vaccine and the threat posed by the disease is crucial at all times, these findings suggest that it may be particularly effective at encouraging uptake during the early stages of rollouts and for novel vaccines and diseases, given that numeracy and risk perceptions did not remain significant predictors of vaccine uptake later in the pandemic.

Our findings offer important evidence that attitudes and intentions towards COVID-19 predict uptake and provide validation for the many studies that have used these measures as a proxy for vaccination uptake [3, 5–8, 10, 11]. In fact, of our respondents who were 65 years or older, 90% of those who reported that they intended to vaccinate had done so by the March 2021 survey. In addition, the present findings also build on prior research exploring characteristics associated with COVID-19 vaccination uptake, which has tended to overlook psychological and behavioral factors [19–21]. Taken together, these findings reinforce the need to develop effective strategies for addressing people’s concerns and negative attitudes towards COVID-19 vaccines in order to increase uptake.

The findings from the present study may also contribute to informing health communication efforts aimed at those least likely to get a COVID-19 vaccine. Around 10% of the respondents in our study both had not been vaccinated at the time of the final survey in March and also indicated that they did not intend to do so in the future. These respondents tended to be younger, had negative views about the COVID-19 vaccines for adults, low trust in healthcare, and preferred to watch and wait before taking action in medical situations where there is clinical equipoise on whether action is necessary. In addition, the most important reasons given by these respondents for not getting a COVID-19 vaccine focused on safety concerns (particularly regarding side effects and the development process), beliefs that COVID-19 is not a serious threat, personal beliefs conflicting with vaccination and distrust of institutions involved with the vaccines.

Our findings are aligned with prior studies on the reasons given by people who are hesitant towards or refuse COVID-19 vaccines [3, 47, 48], and offer an empirical basis for targeting public health messages to those who are least likely to vaccinate and tailoring messages to address their concerns. As these beliefs are often deeply held and traditional models of health communication have been largely ineffective at addressing them [32, 49], we encourage health researchers and communicators to move beyond such traditional models of information provision and instead generate alternative strategies for addressing the concerns of those who are reluctant to get vaccinated. This is particularly important, given that the CDC COVID Data Tracker currently estimates that ~20% of adults in the United States are without a primary dose of a COVID-19 vaccine and 83% have not received an updated booster vaccine [2]. 

One limitation of the study is that the findings rely on the accuracy and consistency of respondents’ self-reported data over the duration of the survey period. Although self-reports have been shown to be highly concordant with healthcare utilization and vaccine records [50, 51], replication of these findings with a method for confirming respondents’ reported vaccine uptake would increase confidence in these findings.

Furthermore, our sample consisted of Veteran and non-Veteran respondents who were unique in being sufficiently motivated and able to complete three online surveys during the pandemic and therefore are not representative of the general U.S. population. The finding that Veteran status did not predict vaccine uptake at either time point was surprising given the efforts and widespread outreach of the U.S. Department of Veterans Affairs in supporting COVID-19 vaccine distribution [52]. However, it is likely that the greater proportion of older adults in the Veteran sample compared to the non-Veteran sample may have limited our ability to observe a significant effect of Veteran status in the full model.

The unique makeup of our sample may also explain why the only early eligibility and demographic factors associated with vaccine uptake in this study were older age and numeracy. Our findings might also differ from prior research as our sample was overrepresented by respondents without many pre-existing health conditions (70% reported ≤ 1 pre-existing health condition), with high health literacy (94% of respondents reported high health literacy), and who identified as non-Hispanic White (78%). For instance, due to the high proportion respondents in our sample who identified as non-Hispanic White, we did not have sufficient power to explore differences across other specific racial and ethnic subgroups, which have been shown in prior studies to be associated with vaccine intentions and uptake [11].

## Conclusion

Despite these limitations, the findings from the present study offer important insights regarding the predictors of vaccine uptake during the early stages of the COVID-19 vaccine rollout in the US, which can help guide health communications and public outreach. In this study, we found that early uptake of COVID-19 vaccines (i.e., by January 2021) was associated with older age, greater numeracy skills, higher COVID-19 risk perceptions, and positive attitudes towards COVID-19 vaccines, while later vaccine uptake (i.e., by March 2021) was characterized by older age, positive attitudes towards COVID-19 vaccines, and intentions to receive the vaccine. Younger age, negative attitudes towards COVID-19 vaccines, low trust in healthcare, and medical minimizing, were significant predictors of being unvaccinated and not wanting to receive a COVID-19 vaccine, as of March 2021. These findings reinforce the need for developing effective strategies for promoting positive attitudes and intentions towards vaccines to promote uptake and highlight the importance of tailoring efforts to address the unique concerns of those who are least likely to get vaccinated. A major strength of our study is that we were able to cover the initial stages of the COVID-19 vaccine distribution. However, given the changes observed between January and March and the unique characteristics of our sample, further studies are needed to re-evaluate the key predictors of vaccine uptake as the rollout progresses and with wider representation, particularly as individuals become eligible for booster vaccines and considering the circulation of novel SARS-CoV-2 variants.

### Electronic supplementary material

Below is the link to the electronic supplementary material.

**Supplementary Material:** Online Supplementary Materials

## Data Availability

The materials and datasets for this study are publicly available at: https://osf.io/63gte/. Citation: Thorpe, A., Fagerlin, A., Scherer, L., Drews, F., Butler, J., Stevens, V., Shoemaker, H., Burpo, N., & Riddoch, M. (2022). *Veterans Experiences during the COVID-19 pandemic (VISION-19)*. 10.17605/OSF.IO/63GTE. The pre-registration protocol for this study is available at: https://aspredicted.org/MKS_HRZ. This manuscript has been uploaded as a pre-print, available at: https://www.medrxiv.org/content/10.1101/2022.04.19.22273818v2.

## Footnotes

1 In our pre-registration (https://aspredicted.org/MKS_HRZ) we erroneously stated that we would expect “lower health literacy, lower numeracy” to be associated with vaccine uptake. This was an error and is therefore corrected in the manuscript.
