# Supplementary material for: Predictors of COVID-19 vaccine uptake: an online three-wave survey study of US adults
Source: BMC Infect Dis. 2024 Mar 12;24:304. doi: 10.1186/s12879-024-09148-9 (PMC10936026; doi:10.1186/s12879-024-09148-9)
Supplement: Supplementary file 1 — Supplementary Material: Online Supplementary Materials [file 12879_2024_9148_MOESM1_ESM.docx]

**Online Supplementary Materials**

**Title:** Predictors of COVID-19 vaccine uptake: An online three-wave survey study of US adults.

This document contains links to an online report describing all the measures included in the analyses, the study pre-registration document, and the project OSF page. A supplemental table showing bivariate correlations and odds ratios for predictor variables and COVID-19 vaccine uptake in January and March of 2021 is also included. The table of contents below can be used for navigation.

Table of Contents

[Links for study measures, pre-registration, and OSF page 2](#_Toc126228536)

[Supplemental Table 3](#_Toc126228537)

# Links for study measures, pre-registration, and OSF page

Link to descriptions of all measures included in the analyses:

<https://rpubs.com/AThorpe/CV19VaxUptakeMeasures>

Link to the study pre-registration document:

<https://aspredicted.org/MKS_HRZ>

Link to the project OSF page:

<https://osf.io/63gte/>

# Supplemental Table

| **Supplemental Table. Bivariate correlations and odds ratios for predictor variables and COVID-19 vaccine uptake in January and March of 2021.** | | | | | | | | | | | | | |
| --- | --- | --- | --- | --- | --- | --- | --- | --- | --- | --- | --- | --- | --- |
|  |  |  | January-2021, COVID-19 vaccine uptake (≥1 dose) | | | | |  | March-2021, COVID-19 vaccine uptake (≥1 dose) | | | | |
|  |  |  | *r* | *P value^*^* |  | OR | *P value* |  | *r* | *P value^*^* |  | OR | *P value* |
| Age |  |  | 0.16 (0.10, 0.22) | <.001 |  | 2.02 (1.13, 3.78) | .021 |  | 0.50 (0.45, 0.55) | <.001 |  | 10.92 (6.76, 18.05) | <.001 |
| Proportion of state with +1 dose |  |  | 0.07 (0.00, 0.13) | .239 |  | 1.09 (0.99, 1.18) | .063 |  | 0.08 (0.01, 0.15) | .070 |  | 0.99 (0.90, 1.10) | .909 |
| Veteran |  |  | 0.10 (0.04, 0.16) | .034 |  | 1.21 (0.80, 1.84) | .379 |  | 0.22 (0.15, 0.29) | <.001 |  | 1.21 (0.80, 1.84) | .366 |
| Total # of pre-existing conditions |  |  | 0.09 (0.03, 0.16) | .018 |  | 1.10 (0.96, 1.26) | .174 |  | 0.17 (0.10, 0.24) | <.001 |  | 1.10 (0.94, 1.28) | .233 |
| Health literacy |  |  | -0.02 (-0.09, 0.04) | .948 |  | 1.09 (0.80, 1.44) | .557 |  | -0.08 (-0.15, -0.01) | .060 |  | 0.96 (0.72, 1.28) | .796 |
| Numeracy |  |  | 0.17 (0.10, 0.23) | <.001 |  | 1.48 (1.20, 1.86) | <.001 |  | 0.18 (0.11, 0.25) | <.001 |  | 0.94 (0.79, 1.13) | .509 |
| Non-Hispanic White |  |  | 0.04 (-0.02, 0.11) | .797 |  | 0.93 (0.58, 1.52) | .774 |  | 0.12 (0.05, 0.19) | <.001 |  | 0.98 (0.60, 1.56) | .917 |
| Worry about getting COVID-19 |  |  | 0.04 (-0.02, 0.10) | .797 |  | 0.84 (0.68, 1.02) | .080 |  | 0.12 (0.05, 0.19) | <.001 |  | 1.21 (0.99, 1.49) | .061 |
| COVID-19 risk perceptions |  |  | 0.11 (0.05, 0.17) | .008 |  | 1.35 (1.03, 1.78) | .029 |  | 0.13 (0.06, 0.20) | <.001 |  | 0.82 (0.62, 1.08) | .163 |
| Emory Vaccine Confidence |  |  | 0.15 (0.08, 0.21) | <.001 |  | 1.00 (0.94, 1.06) | .889 |  | 0.34 (0.28, 0.40) | <.001 |  | 1.01 (0.95, 1.07) | .726 |
| Flu vaccine important |  |  | 0.18 (0.12, 0.24) | <.001 |  | 1.09 (0.81, 1.51) | .581 |  | 0.37 (0.31, 0.43) | <.001 |  | 1.07 (0.83, 1.37) | .606 |
| COVID-19 vaccine important |  |  | 0.20 (0.14, 0.27) | <.001 |  | 1.66 (1.05, 2.66) | .033 |  | 0.43 (0.37, 0.49) | <.001 |  | 1.63 (1.15, 2.34) | .006 |
| COVID-19 vaccine intentions |  |  | 0.20 (0.14, 0.26) | <.001 |  | 1.29 (0.98, 1.74) | .081 |  | 0.42 (0.36, 0.48) | <.001 |  | 1.37 (1.10, 1.72) | .006 |
| Trust in healthcare |  |  | 0.08 (0.01, 0.14) | .135 |  | 0.89 (0.74, 1.09) | .265 |  | 0.25 (0.18, 0.32) | <.001 |  | 1.11 (0.90, 1.36) | .337 |
| (lack of) Belief in science |  |  | -0.15 (-0.21, -0.08) | <.001 |  | 0.87 (0.74, 1.02) | .089 |  | -0.25 (-0.31, -0.18) | <.001 |  | 0.84 (0.72, 0.99) | .041 |
| Belief in conspiracy theories |  |  | -0.11 (-0.17, -0.05) | .008 |  | 1.10 (0.81, 1.47) | .532 |  | -0.25 (-0.32, -0.19) | <.001 |  | 1.03 (0.80, 1.33) | .823 |
| Conservative beliefs |  |  | -0.02 (-0.09, 0.04) | .948 |  | 1.04 (0.92, 1.19) | .500 |  | -0.02 (-0.09, 0.05) | .610 |  | 1.05 (0.91, 1.20) | .514 |
| Maximizing |  |  | 0.05 (-0.01, 0.12) | .473 |  | 0.97 (0.85, 1.11) | .682 |  | 0.14 (0.07, 0.21) | <.001 |  | 1.11 (0.97, 1.27) | .128 |
|  |  |  |  |  |  | N=925, R^2^ Tjur=0.11 | |  |  |  |  | N=758, R^2^ Tjur=0.40 | |

*r* represents the bivariate correlation coefficient. OR represents the odds ratio. 95% confidence intervals (CIs) are shown in parentheses.

*Holm-Bonferroni correction applied
